# Assessing the arrhythmogenic propensity of fibrotic substrate using digital twins to inform a mechanisms-based atrial fibrillation ablation strategy

**DOI:** 10.1038/s44161-024-00489-x

**Published:** 2024-06-18

**Authors:** Kensuke Sakata, Ryan P. Bradley, Adityo Prakosa, Carolyna A. P. Yamamoto, Syed Yusuf Ali, Shane Loeffler, Brock M. Tice, Patrick M. Boyle, Eugene G. Kholmovski, Ritu Yadav, Sunil Kumar Sinha, Joseph E. Marine, Hugh Calkins, David D. Spragg, Natalia A. Trayanova

**Affiliations:** 1 Alliance for Cardiovascular Diagnostic and Treatment Innovation, Johns Hopkins University, Baltimore, MD, USA.; 2 Research Computing, Lehigh University, Bethlehem, PA, USA.; 3 Department of Biomedical Engineering, Johns Hopkins University, Baltimore, MD, USA.; 4 Center for Cardiovascular Biology, University of Washington, Seattle, WA, USA.; 5 Institute for Stem Cell and Regenerative Medicine, University of Washington, Seattle, WA, USA.; 6 Division of Cardiology, Department of Medicine, Johns Hopkins University School of Medicine, Baltimore, MD, USA.

## Abstract

Atrial fibrillation (AF), the most common heart rhythm disorder, may cause stroke and heart failure. For patients with persistent AF with fibrosis proliferation, the standard AF treatment—pulmonary vein isolation—has poor outcomes, necessitating redo procedures, owing to insufficient understanding of what constitutes good targets in fibrotic substrates. Here we present a prospective clinical and personalized digital twin study that characterizes the arrhythmogenic properties of persistent AF substrates and uncovers locations possessing rotor-attracting capabilities. Among these, a portion needs to be ablated to render the substrate not inducible for rotors, but the rest (37%) lose rotor-attracting capabilities when another location is ablated. Leveraging digital twin mechanistic insights, we suggest ablation targets that eliminate arrhythmia propensity with minimum lesions while also minimizing the risk of iatrogenic tachycardia and AF recurrence. Our findings provide further evidence regarding the appropriate substrate ablation targets in persistent AF, opening the door for effective strategies to mitigate patients’ AF burden.

Atrial fibrillation (AF) is the most common heart rhythm disorder and is associated with risk of stroke, heart failure, cardiovascular hospitalization and death^1,2^. AF affects patients’ quality of life and cognitive function^3,4^. Aiming to achieve a cure for AF, catheter-based ablation destroys, regionally, the ability of cardiac tissue to conduct electrical signals, potentially leading to arrhythmia termination. The standard-of-care ablation treatment for AF is pulmonary vein (PV) isolation (PVI), which, by encircling the PVs with lesions, prevents PV ectopic beats from propagating in the atria and inducing AF^5^. However, PVI-only ablation has achieved a success rate of only 59–71% in patients with the persistent form of AF (PsAF)^6,7^. Recent research has demonstrated that this success rate remains unchanged when PVI lesions are shown to be durable^8^. In patients with PsAF, the atria typically remodel and become fibrotic^9,10^. The distributed atrial fibrosis creates a substrate for abnormal electrical propagation by altering local cellular membrane kinetics, causing slow and disconnected signal conduction and giving rise to reentrant electrical waves^11–13^. Attempts to eliminate the arrhythmogenic propensity of the fibrotic substrate by delivering linear ablation lesions across the left atrial (LA) roof and mitral valve isthmus, so that parts of the atria become electrically isolated, or by targeting complex fractionated atrial electrograms (CFAEs), the latter thought to reflect the structural heterogeneity of the substrate, have consistently failed to deliver improved outcomes over PVI alone^6,7,14,15^. Atrial low-voltage areas (LVAs) have also been targeted, with variable success^16,17^. Visualizing the fibrosis distribution on contrast-enhanced magnetic resonance imaging (MRI) (late gadolinium enhancement MRI (LGE-MRI)) before ablation and targeting it by a variety of extra-PVI lesions, typically at the discretion of the operator but not based on mechanistic considerations, has also not achieved superior outcomes to PVI-only ablation^18^. Attempts to directly target rotational activities arising from the PsAF-remodeled substrate^19–22^ have had no effect on improving clinical outcome beyond that of PVI^23^. The failure of these extra-PVI ablation strategies to improve procedure success and decrease redo ablations stems from the fact that there is currently no understanding as to what constitutes an appropriate ablation target in the atrial fibrotic substrate.

Additionally, the uncertainty in determining the extra-PVI targets often results in excessive lesions, increasing the probability of macro-reentrant atrial tachycardia forming around the scar lesion after ablation (that is, iatrogenic atrial tachycardia (iAT)). These iATs are often more refractory to both ablation and drug therapy than AF, thereby requiring frequent cardioversion^24,25^. Excessive ablation may also result in stiff LA syndrome, leading to pulmonary arterial hypertension^26^. These clinical realities underscore the urgent need to develop new personalized approaches for PsAF ablation that account mechanistically for the distributed atrial fibrosis and the resulting heterogenous arrhythmogenic properties, thereby improving the efficacy of the therapy and markedly reducing the need for repeated procedures.

Here we present a combined prospective clinical and personalized mechanistic computational study aimed at comprehensively characterizing the arrhythmogenic properties of the atrial fibrotic substrate in patients with PsAF. Based on the acquired insight, we suggest what set of extra-PVI ablation targets would eliminate substrate arrhythmia propensity in each patient with PsAF with minimum lesion size while also minimizing both the risk of iAT and the potential for redo ablation. For each enrolled patient with PsAF undergoing AF ablation, a personalized atrial computational model (a ‘digital twin’ (DT)) was constructed from the patient’s pre-procedure LGE-MRI scan and was used, in combination with intra-procedural electrogram recordings, to determine mechanistically how best to eliminate the arrhythmogenic propensity of the fibrotic substrate while maximally preserving atrial function. Our results provide new evidence in the ongoing debate regarding what are the appropriate substrate targets for AF ablation in patients with fibrosis and open the door for effective strategies to mitigate patients’ AF burden.

## Results

**Fig. 1 |:**
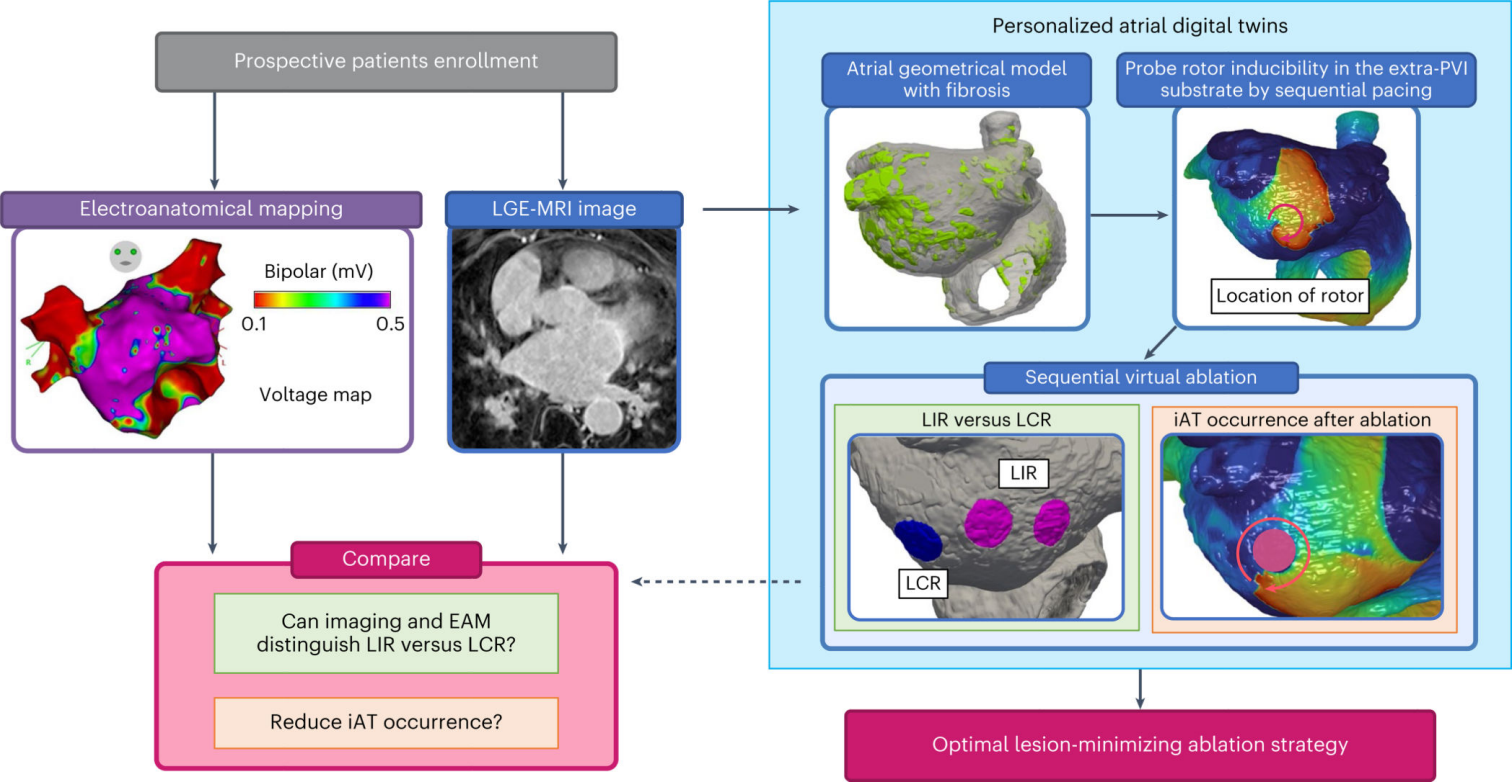
Overview of the combined prospective clinical and personalized DT study. In patients with PsAF enrolled prospectively, LGE-MRI images were acquired before the ablation procedure (blue panel, left). EAM maps were acquired during the procedure (purple panel, left). Right, light blue box, personalized digital twinning. A personalized atrial DT was constructed from LGE-MRI (top left image), and locations capable of sustaining rotors in the extra-PVI substrate were identified (top right image). LIRs, those capable of giving rise to sustained rotors and the preferred ablation targets for non-inducibility, are discerned from LCRs, those that lose their rotor-attracting capabilities when another rotor is eliminated (image within the green panel), and the likelihood of iAT occurrence at ablation locations is investigated (image within the orange panel). FD and FE values and EAM data were analyzed at these locations to uncover the features of LIRs and LCRs and of iAT-inducing locations (cranberry panel, left).

Figure 1 presents an overview of our prospective clinical and DT study. Atrial LGE-MRI scans were acquired from enrolled patients with PsAF undergoing AF ablation (Fig. 1, blue panel, left). High-density global bi-atrial electroanatomical maps (EAMs) were acquired intra-procedurally during sinus rhythm (SR) (Fig. 1, purple panel, left). The LGE-MRI images were used, after processing, to construct personalized computational models (DTs) of the patients’ atria, reflecting their unique fibrosis distribution (Fig. 1, top left image within the light blue box) and the ensuing atrial electrical activity. The arrhythmogenic propensity of the substrate was assessed by stress testing it—that is, by subjecting the DTs to sequential rapid pacing from many sites to probe which locations in the fibrotic distribution outside of PVI can give rise to persistent reentrant electrical activities—that is, rotor-attracting locations (Fig. 1, top right image within the light blue box). Once these substrate locations were identified, they were targeted by virtual ablations in the DT. These were performed sequentially, target by target, or in groups of targets, and in different orders of execution. After each ablation, the inducibility test was repeated in the DTs to assess the arrhythmogenic propensity of the new atrial substrate consisting of native fibrosis (by which we refer to the enhancement on the pre-procedure MRI, which sometimes includes previous ablation scars that have become, over time, indistinguishable from non-ablation fibrosis) and executed lesion(s), and further virtual ablations were performed if new targets were found (that is, if new locations capable of sustaining rotors emerged). The process lasted until complete rotor non-inducibility, from any pacing site, was achieved in the substrate of all DTs. In the course of this sequential probing-and-ablation approach, we identified two types of rotor-sustaining locations in the fibrosis substrate: (1) those capable of giving rise to sustained rotors that needed to be ablated to terminate the activity (termed locations of independent rotors (LIRs)) and (2) those where a sustained rotor was initially inducible but did not form after a lesion was executed at another rotor location elsewhere in the substrate (termed locations of contingent rotors (LCRs)) (Fig. 1, bottom left within the light blue box). LCRs are those that were arrhythmogenic in the native fibrosis substrate but became not arrhythmogenic in the new substrate consisting of native fibrosis plus ablation lesion(s) somewhere in the atria. We also determined the likelihood of iATs occurring around lesions executed at rotor locations (Fig. 1, bottom right image within the light blue box). Uncovering substrate locations that are LCRs so that they are not targeted for ablation and mitigating the risk of iAT are critical steps in preventing excessive ablation while ensuring substrate non-inducibility and durable success of the procedure.

The completion of this approach established a final extra-PVI ‘lesion-minimizing’ set of ablation targets. Next, for each patient, these target locations were analyzed together with (1) electrograms from the global intra-procedure EAM and (2) the LGE-MRI fibrosis distribution, from which maps of fibrosis density (FD) and fibrosis entropy (FE) were constructed (Methods). The analysis aimed to determine whether there are clinically detectable signatures of LIRs and LCRs, as well as of post-ablation iAT propensity, and whether these could provide guidance in achieving a ‘lesion-minimizing’ ablation procedure, separate from the potential benefit of personalized computational modeling (that is, digital twinning). Finally, our ‘lesion-minimizing’ ablation target set was compared to an ablation target set arrived upon using the same methodology but without discerning and sparing the LCR, as done in our previous study^27^. The latter study was the first proof-of-concept prospective study demonstrating the utility of personalized atrial DTs in guiding ablation, with a high procedure success rate. The comparison between the lesion strategy in the current study and the one in this previous study^27^ highlighted the myocardium-preserving benefits of the current approach.

### Extra-PVI rotor-sustaining locations in fibrotic substrate

**Extended Data Fig. 1 |:**
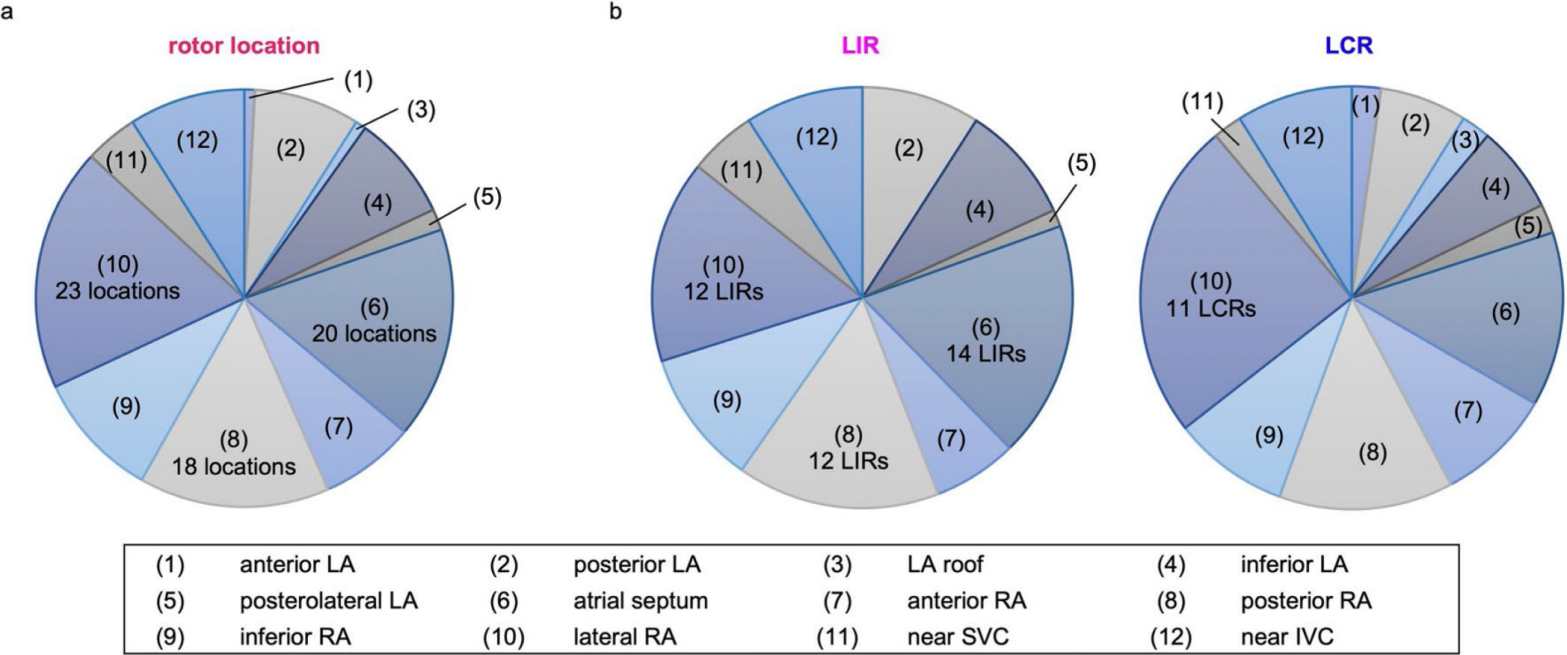
Distribution of extra-PVI rotor locations in the 12 atrial regions. **a:** Rotor locations. **b:** LIRs and LCRs.

For the 35 consecutive patients with PsAF enrolled in this prospective study, 26 had personalized atrial DTs constructed (see Methods for exclusion). In five patients, the fibrotic substrate did not harbor reentrant activities outside PVI. In each DT constructed for the 26 patients, we achieved arrhythmia non-inducibility from any pacing location after the ablation strategy described above (see Methods for additional details). In total, 122 rotor locations were identified in the substrate outside of the PVI lines in 21 patients; there were 34 in the LAs and 88 in the right atria (RAs). Dividing each patient’s bi-atrial DT into 12 regions (Methods), we found that locations capable of sustained rotors were predominantly located in the RA lateral wall (Extended Data Fig. 1a). These results have important implications, as they indicate that the RA is critically involved in the mechanisms of arrhythmogenesis in PsAF. The RA has been largely ignored in many previous clinical studies of the atrial fibrotic substrate^18,28,29^, with the LA generally thought to be the main target region for rotor detection and modulation^7,16,17,30–32^.

**Fig. 2 |:**
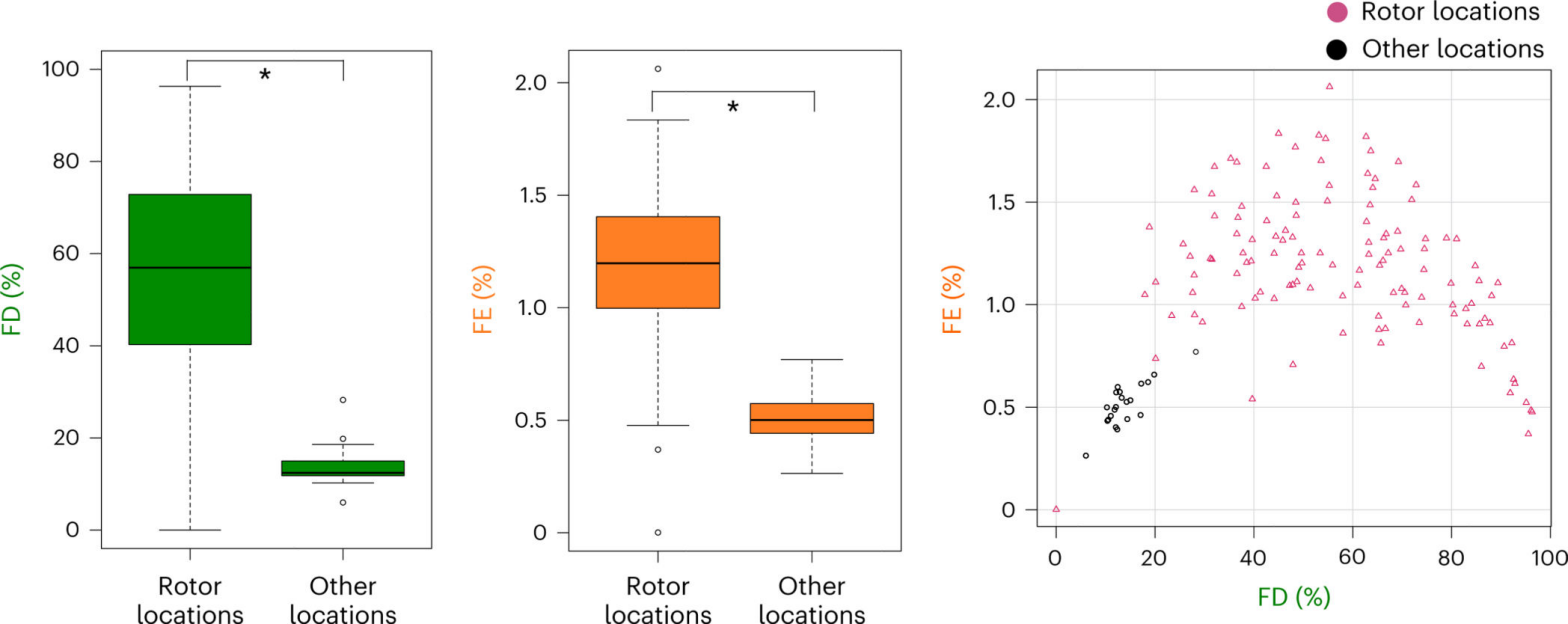
Fibrosis distribution at rotor locations identified in DT substrates. Left, FD values in the substrate (rotor locations, *n* = 122; other locations, *n* = 21; independent samples; data are presented as median (Q1–Q3) with minimum and maximum; *P* = 1.22 × 10^−12^; two-sided Mann–Whitney *U*-test without adjustments). *P < 0.05. Middle, FE values in the substrate (rotor locations, *n* = 122; other locations, *n* = 21; independent samples; data are presented as median (Q1–Q3) with minimum and maximum; *P* = 1.67 × 10^−11^; two-sided Mann–Whitney *U*-test without adjustments). Right, FD and FE values at rotor locations (cranberry triangles) and other locations (black circles).

Analyzing the fibrosis distribution, both FD and FE were significantly higher at substrate locations capable of sustaining rotors than at those that could not (Fig. 2). FD was 57% (41–73) versus 12% (12–15) (*U* = 2,527, *P* < 0.001), and the area under the curve (AUC) for predicting rotor locations was 0.99 (0.97–1), with a sensitivity of 0.975 and a specificity of 0.952. FE was 1.20% (1.00–1.40) versus 0.50% (0.44–0.57) (*U* = 2,462, *P* < 0.001), and the AUC for predicting rotor locations was 0.96 (0.93–0.99), with a sensitivity of 0.902 and a specificity of 1.000. Fibrosis distributions with similar FE and FD have been found to be border zones of fibrosis where potential interdigitation of fibrotic and non-fibrotic tissue takes place, setting up the stage for reentry formation^27,33^. Our analysis here demonstrated that both LIRs and LCRs were within the boundary zones of fibrosis.

### Classification of LIRs and LCRs in the personalized DTs

Overall, among the 122 locations of rotor induction in all atrial substrates, there were 77 LIRs and 45 LCRs. No significant difference was observed between LA and RA in the ratio of number of LIRs to the total number of rotor-inducing locations (71% versus 60%; *P* = 0.306), indicating that chamber anatomical differences do not have causal relationship to the proportion of LIRs versus LCRs. Additionally, LIRs were found most frequently at the atrial septum, whereas LCRs were predominantly at the RA lateral wall (Extended Data Fig. 1b). These results indicate that the RA (especially the atrial septum) is critically involved in the mechanism of reentry inducibility in patients with fibrotic remodeling.

**Fig. 3 |:**
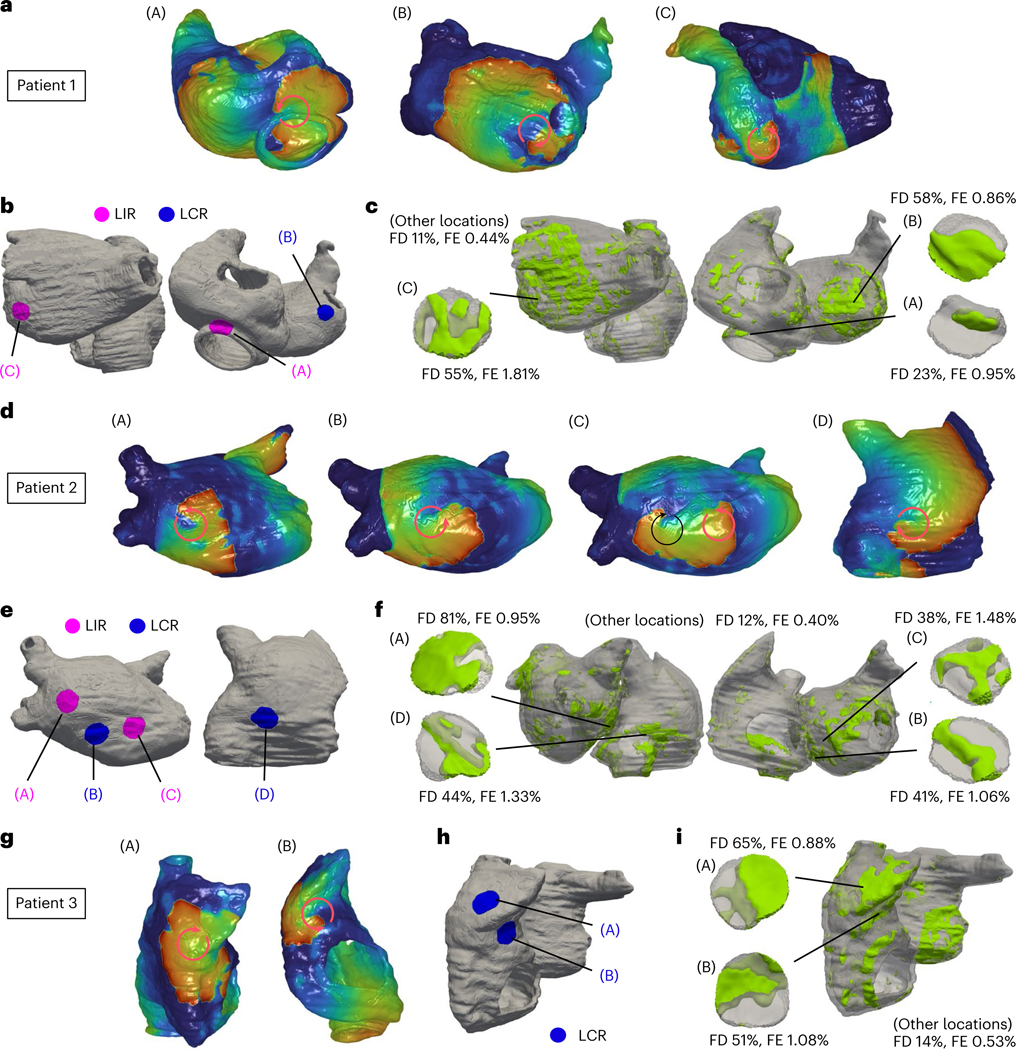
Personalized DTs and ablation strategy for three representative patients (patients 1–3). **a**,**d**,**g**, Identification of rotor location. The red color and the blue color in DTs indicate wave front and wave tail of reentrant propagation (circular arrows), respectively. **b**,**e**,**h**, Classification of rotor locations into LIRs and LCRs. **c**,**f**,**i**, Fibrosis distribution (in light green).

Three examples of rotor classification are shown in Fig. 3. In the first patient, after sequential pacing from the 40 bi-atrial locations, three extra-PVI rotor locations were identified in the DT, marked as (A), (B) and (C) (Fig. 3a). Ablating only rotor location (A) or location (B) did not affect the other rotors ((C) and (B) or (C) and (A), respectively) and did not cause the appearance of rotors at new locations. Ablating only rotor location (C) did not affect rotor (A) occurrence and did not lead to the appearance of rotors at new locations; however, it caused the rotor disappearance at location (B). Therefore, rotor locations (A) and (C) were classified as LIRs, whereas location (B) was classified as LCR (Fig. 3b); ablating these two LIRs resulted in substrate non-inducibility. The FD and FE at rotor locations (A)–(C) are presented in Fig. 3c. In the first patient, both the FD and FE at rotor locations are higher than those at other locations.

In the second case, sequential pacing in the atrial DT induced rotors at three extra-PVI locations: (A), (B) and (C) (Fig. 3d). Ablating only rotor location (A) or location (B) or location (C) did not affect the occurrence of the other rotors and did not lead to the appearance of rotors at new locations. Ablation at both locations (A) and (B) did not affect the rotor occurrence at location (C) and did not lead to appearance of rotors at new locations, whereas ablating both locations (B) and (C) did not affect the rotor occurrence at location (A) but led to the rotor appearance at a new (emergent) location (D). Therefore, rotor locations (A) and (C) were classified as LIRs, whereas locations (B) and (D) were classified as LCRs (Fig. 3e). Ablating both locations (A) and (C) achieved non-inducibility of the substrate. The FD and FE at locations (A)–(D) are presented in Fig. 3f. In the second patient, the mean FD at LIRs was higher than that at LCRs, and the mean FE was similar.

The DT of the third patient uncovered two extra-PVI rotor locations in the substrate: (A) and (B) (Fig. 3g). When one of these locations was ablated, the other location was no longer capable of sustaining rotors, and the substrate became non-inducible. Thus, both locations were classified as LCRs. However, to render the substrate non-inducible, in such cases where these are no LIRs, one of the LCRs needed to be ablated (Fig. 3h). As shown in Fig. 3i, in this third patient, both FD and FE values were higher at the two LCRs than those at other locations.

**Fig. 4 |:**
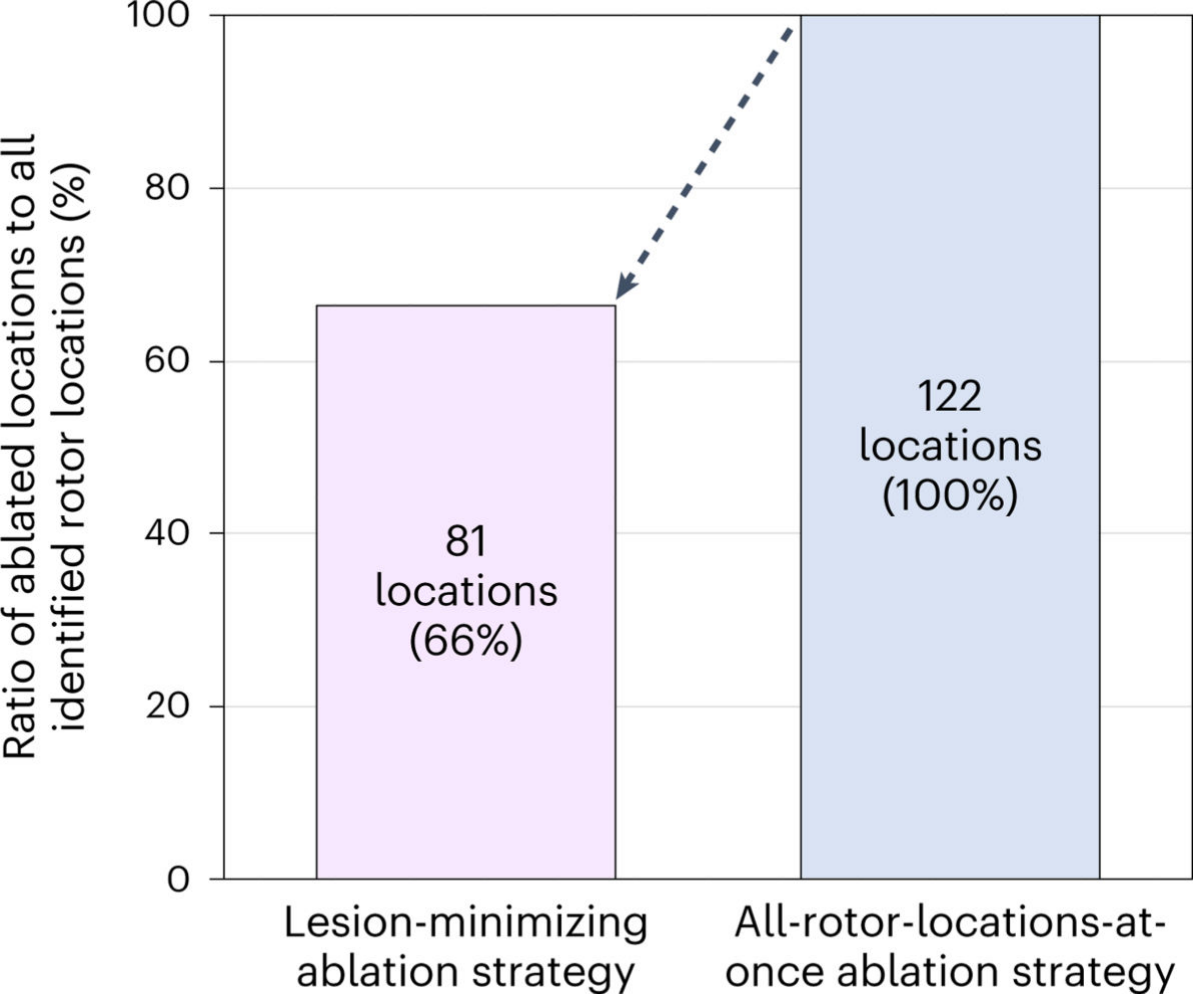
Minimizing ablation lesions with the new ablation strategy. Ratio of the number of ablated rotor locations to that of identified rotor locations for the lesion-minimizing ablation strategy versus that for the all-rotor-locations-at-once ablation strategy.

We compared, in the personalized DTs, our ‘lesion-minimizing’ strategy to an all-rotor-locations-at-once ablation strategy^27^, in which rotor types were not discerned, and all rotor locations were ablated. In the examples above, in patient 1, the latter ablation strategy involved targeting all three rotor locations (A)–(C), after which substrate non-inducibility was achieved. In patient 2, after targeting the three rotor locations (A)–(C) and repeating the inducibility test, there was an emergent rotor location (D). Ablating also (D), for a total of four lesions (versus two lesions in our new strategy), achieved substrate non-inducibility. In the third case, patient 3, as the two LCRs were the only activity that arose in the substrate, our strategy resulted in one lesion versus two otherwise. Overall, among 122 identified rotor locations in all bi-atrial DTs, our strategy targeted 81 rotor locations—that is, 66% of all rotor locations—achieving substrate non-inducibility (Fig. 4). This result indicates that our new substrate ablation reduced the number of target locations by 34% over the entire DT patient cohort.

### Fibrosis burden and rotor-attracting locations in atrial DTs

**Fig. 5 |:**
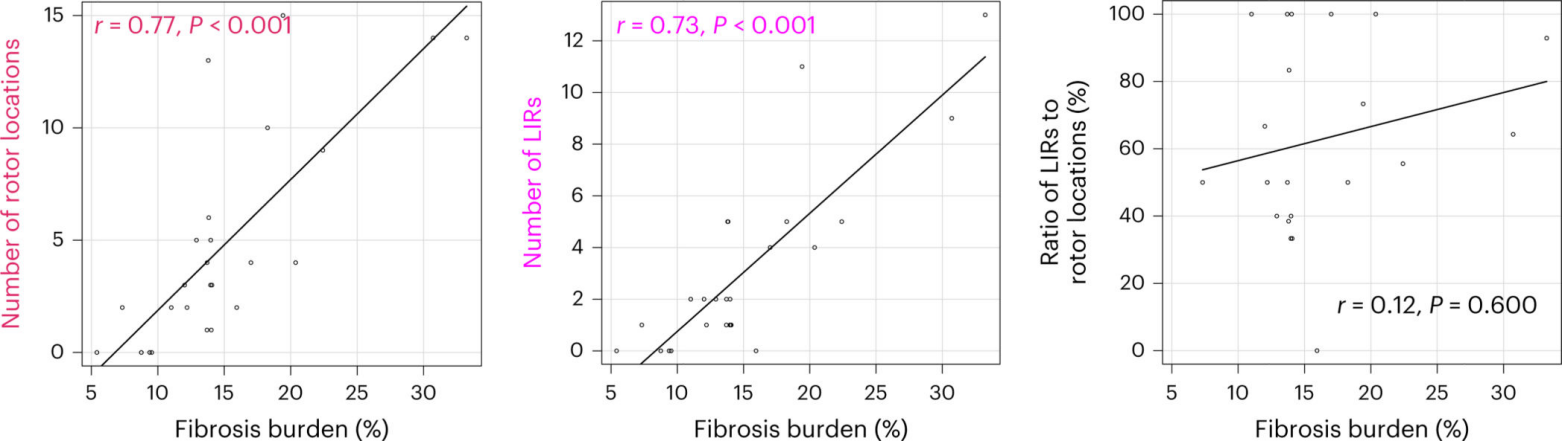
Relationship between fibrosis burden and the number of rotor locations. Left, strong correlation between fibrosis burden and number of rotor locations (*P* = 0.000005; two-sided Spearman’s rank correlation test). Middle, strong correlation between fibrosis burden and number of LIRs (*P* = 0.00003; two-sided Spearman’s rank correlation test). Right, no correlation between fibrosis burden and the ratio of the number of LIRs to that of rotor locations (*P* = 0.600; two-sided Spearman’s rank correlation test).

Previous research reported that patients with PsAF have more atrial fibrosis and larger atria than patients with paroxysmal AF, both features thought to relate to the capability of the PsAF substrate to sustain reentrant drivers^10,34^. In the present study, we found that both the number of rotor locations and the number of LIRs were correlated with global fibrosis burden (amount) rather than atrial volume; for number of rotor locations, *P* < 0.001 versus *P* = 0.609 for fibrosis amount versus atrial volume; for number of LIRs, *P* < 0.001 versus *P* = 0.589 (Fig. 5). However, we found that fibrosis burden had no determinants among clinical characteristics (Supplementary Table 1), indicating that fibrosis burden, as determined by LGE-MRI, is alone an important contributor to the substrate’s ability to sustain rotors. Furthermore, the ratio of the number of LIRs to that of all rotor locations was not correlated with atrial fibrosis burden or with atrial volume (*P* = 0.600 and *P* = 0.758). This result indicates that the substrate’s likelihood to contain LIRs is not determined by what percent of the atrial myocardium has remodeled into fibrosis.

### Relationship of LIR to LGE and intra-atrial electrograms

**Fig. 6 |:**
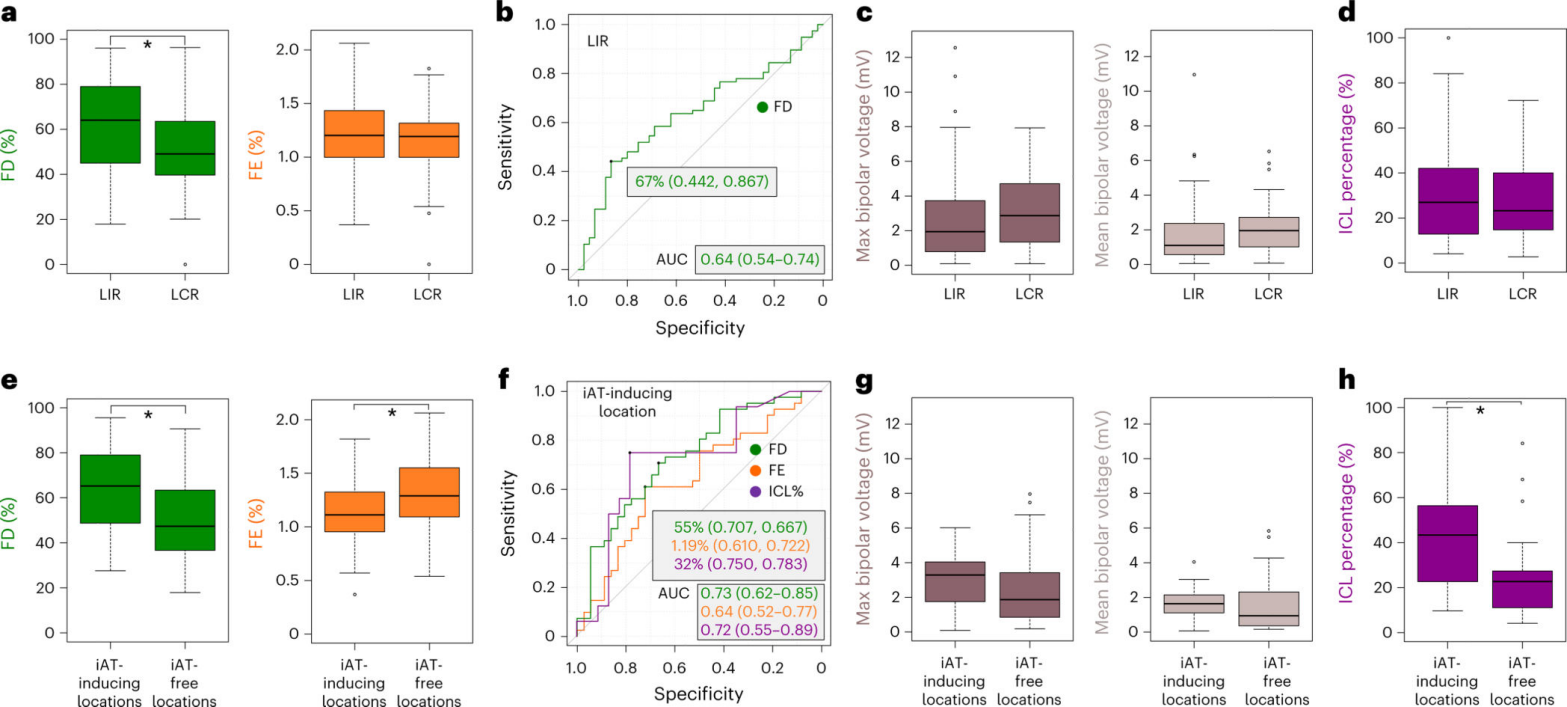
Relationship between types of rotor locations and (1) fibrosis distribution and (2) intra-atrial electrograms. **a**, Comparison of FD and FE values at LIRs and LCRs (LIRs, *n* = 77; LCRs, *n* = 45; independent samples; data are presented as median (Q1–Q3) with minimum and maximum; *P* = 0.011 and *P* = 0.467, respectively; two-sided Mann–Whitney *U*-test without adjustments). *P < 0.05. **b**, ROC curve for predicting LIRs, with the FD cutoff value, the sensitivity and specificity values, and AUC (green). **c**, Comparison of the maximum (brown) and mean (beige) of bipolar voltage at LIRs and LCRs (LIRs, *n* = 48; LCRs, *n* = 29; independent samples; data are presented as median (Q1–Q3) with minimum and maximum; *P* = 0.172 and *P* = 0.127, respectively; two-sided Mann–Whitney *U*-test without adjustments). **d**, Comparison of ICL percentage (purple) at LIRs and LCRs (LIRs, *n* = 41; LCRs, *n* = 24; independent samples; data are presented as median (Q1–Q3) with minimum and maximum; *P* = 0.591; two-sided Mann–Whitney *U*-test without adjustments). **e**, Comparison of FD and FE values at iAT-inducing locations and iAT-free locations (iAT-inducing locations, *n* = 41; iAT-free locations, *n* = 36; independent samples; data are presented as median (Q1–Q3) with minimum and maximum; *P* = 0.00033 and *P* = 0.032, respectively; two-sided Mann–Whitney *U*-test without adjustments). **f**, ROC curves for predicting iAT occurrence, with the cutoff value, the sensitivity and specificity values, and AUC of FD, FE and ICL (green, orange and purple, respectively). **g**, Comparison of the maximum (brown) and mean (beige) of bipolar voltage at iAT-inducing locations and iAT-free locations (iAT-inducing locations, *n* = 21; iAT-free locations, *n* = 26; independent samples; data are presented as median (Q1–Q3) with minimum and maximum; *P* = 0.215 and *P* = 0.127, respectively; two-sided Mann–Whitney *U*-test without adjustments). **h**, Comparison of ICL percentage (purple) at iAT-inducing locations and iAT-free locations (iAT-inducing locations, *n* = 16; iAT-free locations, *n* = 23; independent samples; data are presented as median (Q1–Q3) with minimum and maximum; *P* = 0.021; two-sided Mann–Whitney *U*-test without adjustments).

FD values at LIRs were significantly higher than those at LCRs: 64% (45–79) versus 49% (40–64) (*U* = 2,213, *P* = 0.011) (Fig. 6a). We found that an FD cutoff value of 67% distinguished LIRs from LCRs with 60% accuracy; the AUC and the sensitivity and specificity values are shown in Fig. 6b. However, FE values at LIRs and LCRs were similar: 1.20% (1.00–1.43) versus 1.19% (1.00–1.32) (*U* = 1,870, *P* = 0.467) (Fig. 6a). These results reveal that the local density of fibrosis, rather than its local heterogeneity, determines whether the rotors induced at that location would have the power to persist, even in a new post-ablation substrate.

A total of 77 rotor-attracting locations having intra-atrial electrograms recorded during SR were compared, of which 48 were LIRs and 29 were LCRs. The bipolar voltage at those locations was not decreased (it is commonly expected to decrease <0.5 mV in areas of abnormal tissue^31^); the maximum and mean voltage were 2.57 (1.03–4.10) mV and 1.55 (0.68–2.68) mV, respectively. Both the maximum and mean of the bipolar voltage were lower in LIRs than in LCRs, although the difference had no statistical significance: 1.95 (0.79–3.58) mV versus 2.87 (1.34–4.71) mV for the maximum voltage (*U* = 566, *P* = 0.172) and 1.10 (0.60–2.33) mV versus 1.95 (1.01–2.72) mV for the mean voltage (*U* = 550, *P* = 0.127) (Fig. 6c). Next, fractionated signal area in atrial muscle (FAAM) potentials (Methods) were investigated at 65 rotor-attracting locations among 77 total, after excluding locations with signals of poor quality for FAAM identification; of these, 41 were LIRs and 24 were LCRs. The interval confidence level (ICL) percentage of the FAAM potentials was higher in LIRs than in LCRs, but it did not reach statistical significance: 27% (13–42) versus 23% (16–36) (*U* = 532, *P* = 0.591) (Fig. 6d). Additionally, the FAAM potentials were not determined by the local FD value, which was significantly correlated with LIRs (Supplementary Table 2). These results suggest that distinguishing LIRs using only endocardial intra-procedural atrial signals might be difficult.

### Occurrence of iAT by ablation at rotor-attracting locations

As outlined above, scar-related macro-reentrant atrial tachycardia (that is, iAT) can occur around lesions after ablation; iATs have been frequently reported after ablation in the atrial substrate^24,25^. We, therefore, determined the likelihood of iATs occurring around lesions executed at rotor locations in the atrial DTs (Fig. 1, blue box on the right, bottom right panel). To exclude the potential influence of neighboring lesions on iAT formation, only the 78 rotor locations identified in the native fibrotic substrate were investigated; one location was excluded because its lesion ended up being in contact with a non-conductive barrier, eliminating the possibility of iAT. Of these 77 rotor locations, 26 were in the LAs and 51 were in the RAs. Overall, 41 lesions (53%) led to iATs; these iATs could be eliminated by an ablation line connecting the lesion to the nearest non-conductive obstacle, as previously done^27^. The ratio of the number of iATs to the total number of lesions had no significant difference between LA and RA (54% versus 53%; *P* = 1). iATs were most frequently observed at the atrial septum. These results indicate that iATs could occur with equal probability in RA and LA. This result has important implications, as the LA is often ablated for substrate modification^7,16–18,25^, which, according to our findings, has a high probability of iAT. Moreover, no significant difference was observed in the distance from the closest non-conductive barriers to iAT-inducing lesions and to lesions that did not result in iAT: 21.4 (13.1–31.1) mm versus 18.7 (12.2–30.1) mm (*U* = 761, *P* = 0.822). This suggests that iAT does not depend on the width of the anatomical isthmus formed between the lesion and the adjacent non-conductive barrier through which the tachycardia wave front propagates.

**Extended Data Fig. 2 |:**
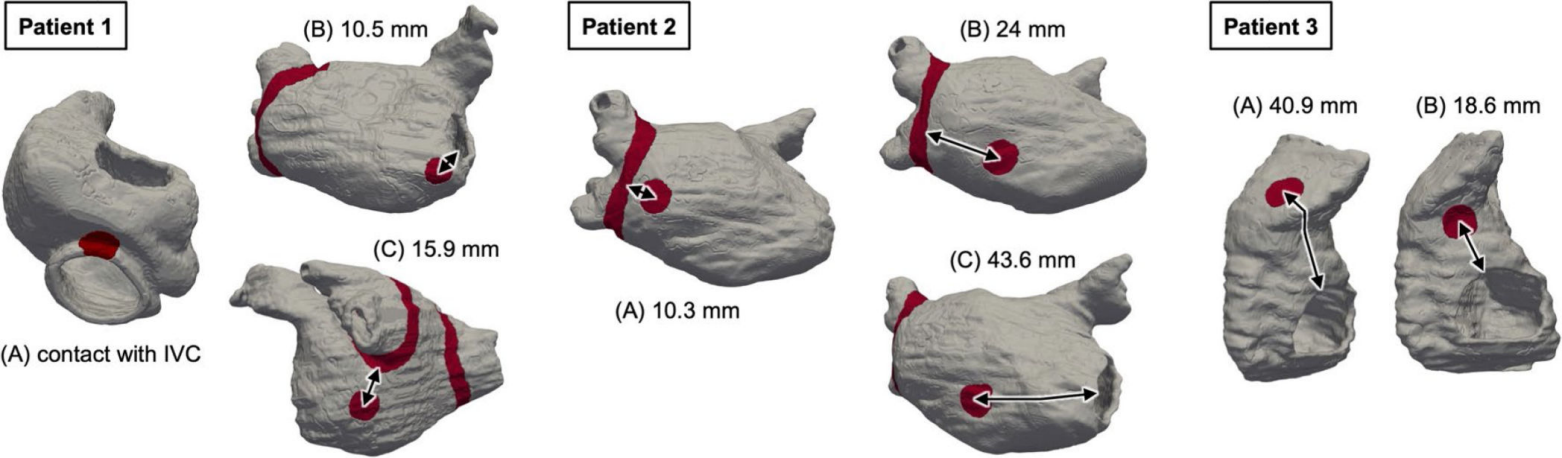
Ablation lesions in three representative DTs (Patients 1–3). Distance between ablated rotor location (red circles) and non-conductive barriers including PVI lines (red lines) is shown with black arrows.

For example, in the DT of patient 1 in Fig. 3, the lesion at rotor location (A) could not lead to iAT because it made contact with the inferior vena cava, a non-conductive barrier. Ablating at rotor location (C), however, resulted in iAT. In the DT of patient 2, all three rotor locations ((A)–(C)) developed iATs after ablation. For patient 3, both lesions at rotor locations (A) and (B) did not induce iAT. The distance from these locations to the closest non-conductive barrier are presented in Extended Data Fig. 2.

### Relationship of iAT to LGE and intra-atrial electrograms

**Extended Data Fig. 3 |:**
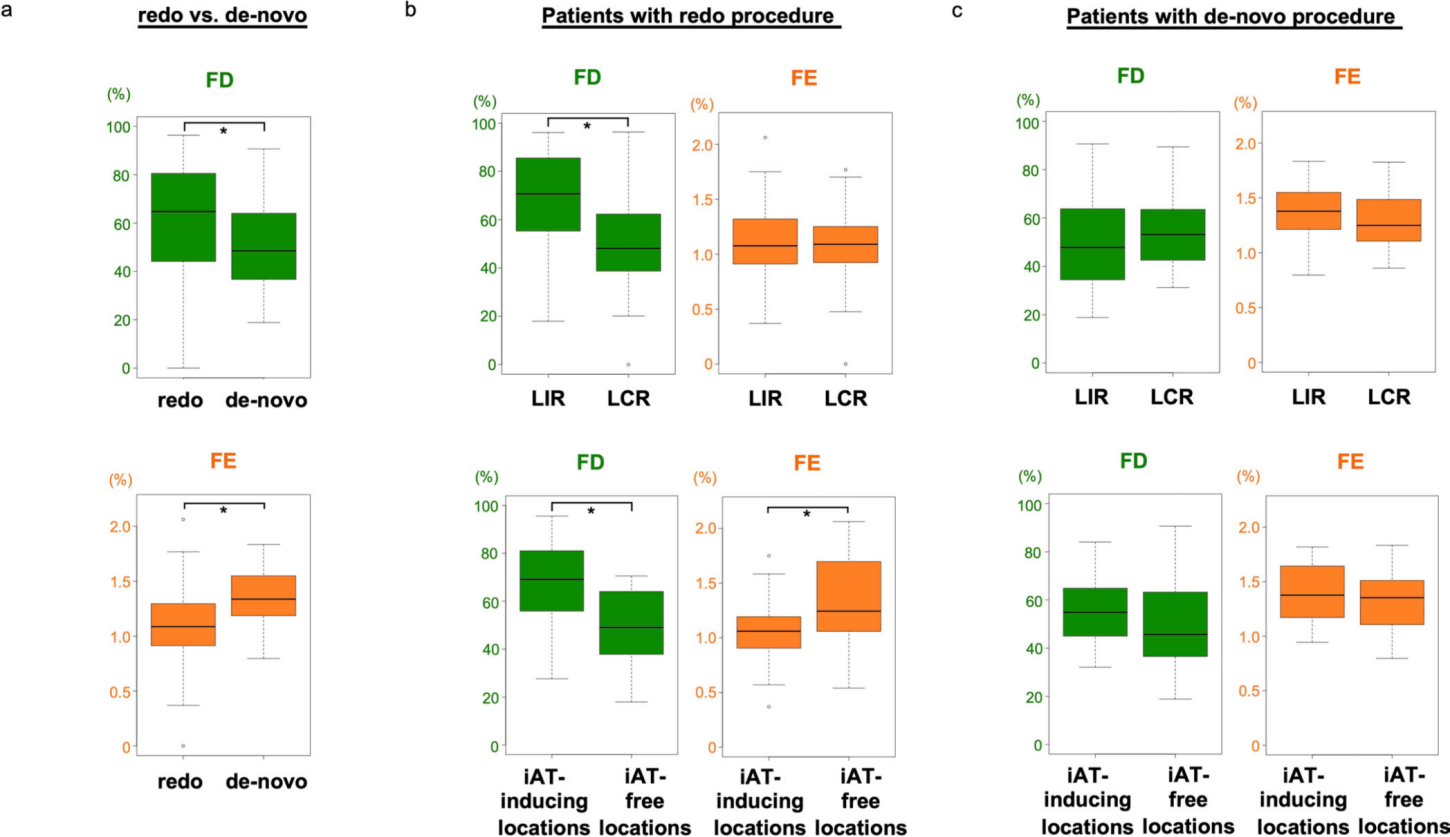
Difference in fibrosis distribution between redo and de novo procedures. **a**: Comparison of FD (green) and FE (orange) values at rotor locations in patients with redo and de-novo procedures; 65% (44–80) vs. 49% (37–64) for FD (redo, n = 74; de-novo, n = 48; independent samples; data are presented as median (Q1-Q3) with min and max; U = 2300, p = 0.006; two-sided Mann-Whitney U test without adjustments); 1.09% (0.91–1.28) vs. 1.34% (1.20–1.54) for FE (redo, n = 74; de-novo, n = 48; independent samples; data are presented as median (Q1-Q3) with min and max; U = 948, p = 0.000015; two-sided Mann-Whitney U test without adjustments). *p < 0.05. **b**: The nine of the 13 patients with redo procedure included in the analysis had 74 extra-PVI rotors in total. The FD was significantly higher at LIRs than LCRs; 71% (57–85) vs. 48% (39–60) (LIRs, n = 46; LCRs, n = 28; independent samples; data are presented as median (Q1-Q3) with min and max; U = 955, p = 0.0004; two-sided Mann-Whitney U test without adjustments). The FE at LIRs was comparable to that at LCRs; 1.08% (0.91–1.32) vs. 1.09% (0.93–1.25) (LIRs, n = 46; LCRs, n = 28; independent samples; data are presented as median (Q1-Q3) with min and max; U = 645, p = 0.996; two-sided Mann-Whitney U test without adjustments). Regarding iAT, the FD at the former was significantly higher than that at the latter; 69% (56–81) vs. 49% (38–62) (iAT-inducing locations, n = 29; iAT-free locations, n = 14; independent samples; data are presented as median (Q1-Q3) with min and max; U = 319, p = 0.002; two-sided Mann-Whitney U test without adjustments). Moreover, the FE was significantly lower at the former than the latter; 1.06% (0.91–1.19) vs. 1.24% (1.10–1.67) for FE (iAT-inducing locations, n = 29; iAT-free locations, n = 14; independent samples; data are presented as median (Q1-Q3) with min and max; U = 115, p = 0.022; two-sided Mann-Whitney U test without adjustments). The remaining 4 patients with redo procedure did not have extra-PVI targets. *p < 0.05. **c**: For patients with de-novo procedures, the 12 out of 13 patients had 48 extra-PVI rotors in total. Both the FD and FE at LIRs were comparable to those at LCRs; 48% (34–64) vs. 53% (43–64) for FD; 1.38% (1.21–1.55) vs. 1.25% (1.11–1.49) for FE (LIR, n = 31; LCRs, n = 17; independent samples; data are presented as median (Q1-Q3) min and max; U = 238, p = 0.594 and U = 305, p = 0.381, respectively; two-sided Mann-Whitney U test without adjustments). Similarly, among 34 rotor locations in the extra-PVI substrate, both the FD and FE at the former were also comparable to those at the latter; 55% (45–65) vs. 46% (37–62) for FD; 1.38% (1.21–1.63) vs. 1.35% (1.13–1.50) for FE (iAT-inducing locations, n = 12; iAT-free locations, n = 22; independent samples; data are presented as median (Q1-Q3) with min and max; U = 167, p = 0.217 and U = 151, p = 0.511, respectively; two-sided Mann-Whitney U test without adjustments). However, compared to the sample size of rotor locations identified in patients with redo procedures, the smaller sample size of those identified in patients with the de novo procedures makes it difficult to interpret.

FD values were significantly higher at iAT-inducing lesion locations than at iAT-free locations in the DTs: 65% (49–79) versus 47% (37–63) (*U* = 1,083, *P* < 0.001). Notably, FE was significantly lower at iAT-inducing locations: 1.11% (0.95–1.32) versus 1.29% (1.10–1.54) (*U* = 528, *P* = 0.032) (Fig. 6e). Cutoff values of the FD and FE for predicting iAT were 55% and 1.19% (with 69% and 66% accuracy, respectively); AUCs and sensitivity and specificity values are shown in Fig. 6f. These results underscore that rotor locations with higher FD but lower FE, such as in compact fibrosis islands, would be the ones most likely to facilitate iAT formation. Extended Data Fig. 3 presents results of DTs broken down by redo and de novo patients, demonstrating that DTs of redo patients are more likely to have LIRs and post-ablation iATs.

**Extended Data Fig. 4 |:**
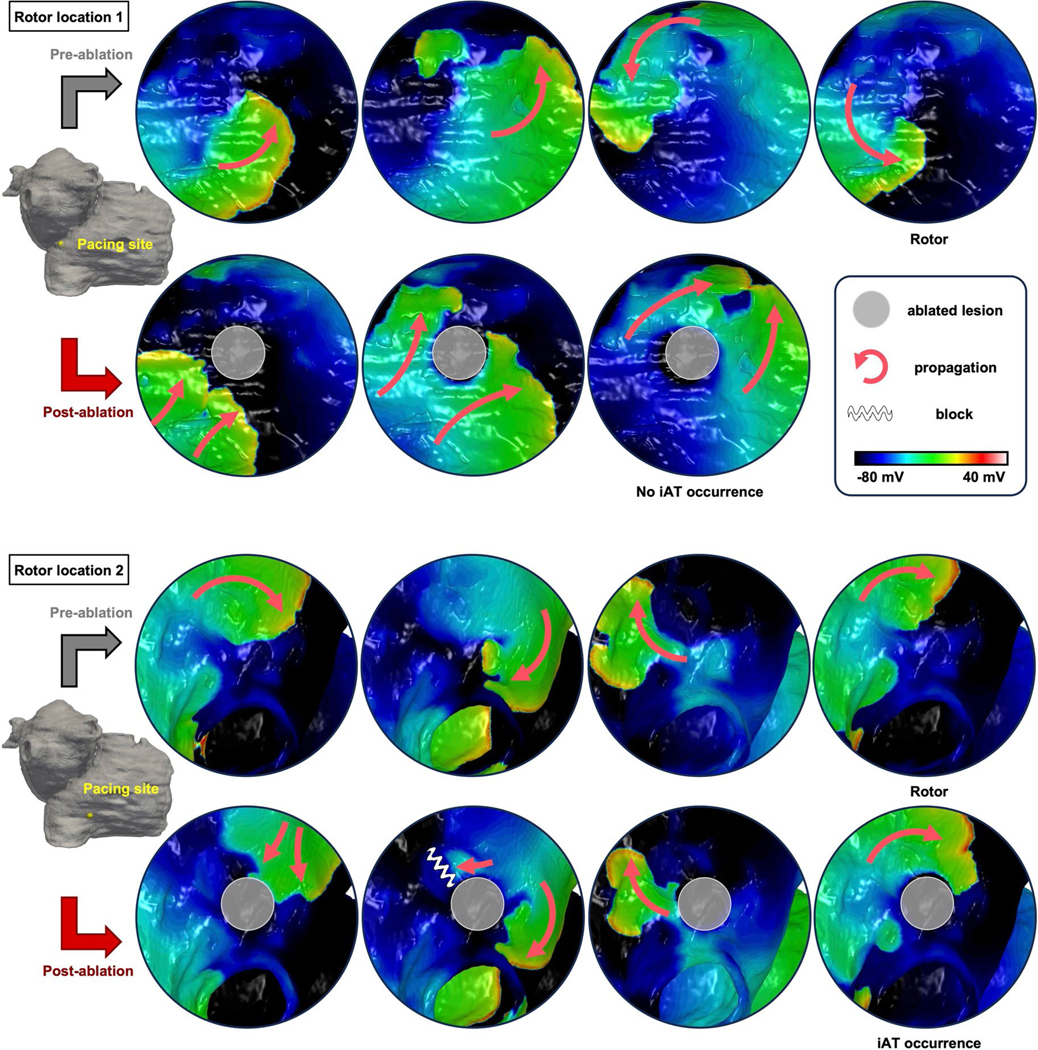
Difference in iAT occurrence at ablated rotor locations in one patient. *Top panel*: An induced rotor with counterclockwise propagation (*upper row*), and a planar propagation around the lesion without iAT occurrence. Pacing sites are the same pre- and post-ablation (*lower row*). *Bottom panel*: Another induced rotor with clockwise propagation (*upper row*), and iAT occurrence with clockwise macro-reentry around the lesion following a unidirectional conduction block at the vicinity of the lesion (*lower row*).

Two rotor-attracting locations in the same patient, of which one resulted in iAT after ablation and the other not, are shown in Extended Data Fig. 4. One was associated with pacing-induced near-planar propagation without slow conduction around the lesion, but the other resulted in the induction of macro-reentry around the lesion after unidirectional block in an area adjacent to the lesion. The location resulting in post-ablation iAT had higher FD and lower FE than the other location (74% versus 50% and 1.17% versus 1.20%, respectively). This indicates that the presence of a compact patch of fibrosis next to the ablated area creates conditions for unidirectional block and reentry around the lesion.

The inter-procedure atrial electrograms during SR were obtained at a total of 47 rotor locations: 21 iAT-inducing locations and 26 iAT-free locations after ablation. There was no difference in bipolar voltage between two locations: 3.29 (1.75–4.04) mV versus 1.87 (0.86–3.38) mV for the maximum voltage (*U* = 332, *P* = 0.215) and 1.64 (1.11–2.14) mV versus 0.94 (0.40–2.19) mV for the mean voltage (*U* = 345, *P* = 0.127) (Fig. 6g). However, it is notable that, among 39 locations with appropriate identification of FAAM ICL—16 iAT-inducing locations and 23 iAT-free locations—the FAAM ICL was significantly higher in the former locations than in the latter: 43% (28–56) versus 23% (11–27) (*U* = 266, *P* = 0.021) (Fig. 6h). For predicting iAT occurrence, the FAAM ICL cutoff value was 32% (with 77% accuracy); AUC, sensitivity and specificity are shown in Fig. 6f. This result suggests that both the local FE value and the FAAM potentials are indicative of the post-ablation iAT-inducing locations, even though bipolar voltage is not. However, multivariate linear regression analysis did not identify FE as a determinant of FAAM potentials. This suggests that the latter do not necessarily arise in local compact fibrosis (low FE value), even though both FE and FAAM ICL local values have significant correlation with iAT (Supplementary Table 2).

## Discussion

In this combined prospective clinical and personalized computational study, we characterized the arrhythmogenic properties of the atrial fibrotic substrate in patients with PsAF with fibrosis and determined the extra-PVI ablation targets that eliminate arrhythmia propensity in each patient with minimum lesions while also minimizing the risk of redo procedure. Using digital replicas of the patients’ atria (bi-atrial DTs, reconstructed from the patients’ clinical MRI scans), we uncovered that not all substrate lesions capable of giving rise to sustained rotors are made equal. Some locations (LCRs) lose their rotor-attracting capabilities when another of the patient-specific rotor locations is targeted by ablation. Other locations (LIRs) persist in forming rotors regardless, and tissue there needs to be ablated to eliminate the propensity to arrhythmia. Furthermore, we found that the substrate’s electrophysiological response to executed lesions also differs: a new scar-related macro-reentrant tachycardia (iAT) emerges in some cases, whereas, in others, it does not. Our DT technology established where additional ablation lines should be executed to prevent iAT from occurring. Using this new substrate assessment strategy, we ascertained the minimum lesion set that eliminates the arrhythmogenic propensity of the substrate while maximally preventing atrial function.

From the imaging and intra-procedural EAM data, we discovered that, although all rotor-attracting locations were in border zones of fibrotic remodeling, locations there with locally denser fibrosis (that is, locations with more fibrosis than myocardium in close vicinity) are more likely to be LIRs (FD values there were significantly higher than those at LCRs). We established the FD cutoff value that defined a location of high likelihood to be an LIR. We also found that iAT-inducing locations have higher FD, lower FE (spatially compact rather than patchy local fibrosis distribution) and higher FAAM ICL (locally slow conduction) values than iAT-free locations. Our results rectify the poor understanding of the atrial arrhythmogenic substrate and add valuable new evidence in the perennial debate in AF management regarding what are the appropriate substrate targets for extra-PVI AF ablation in patients with fibrosis. The analysis of the signal enhancement in the pre-procedure clinical images presented here allows to propose, by non-invasive means, locations of high likelihood to be appropriate ablation targets, which can then be tested, validated and augmented pre-procedurally in the atrial DTs. We, thus, establish an optimal substrate ablation approach guided by the patient’s atrial DT, opening the door for an effective strategy to mitigate patients’ AF burden and preserve their atrial function.

Our study uses bi-atrial DTs to explore the characteristics of the arrhythmogenic substrate and to propose an optimal approach to determining, pre-procedurally, the extra-PVI ablation targets for PsAF. In previous research, the LA was the key target for extra-PVI ablation in PsAF. Ablation strategies have been evaluated on LA but not on RA; strategies include linear ablation^7^, CFAE ablation^7^, vein of Marshall ethanol infusion^32^, LGE-MRI–guided fibrosis ablation^18^, LA LVA ablation^16,17,31^ and posterior wall isolation^30^. However, it is widely recognized that AF is not an LA disease. It results from inflammation^35–37^, including obesity, hypertension and ischemia, leading to myocardium remodeling in both LA and RA^38^. Moreover, research has suggested that RA is also arrhythmogenic: a study monitoring AF cycle length concluded that the RA was driving AF in about 20% of PsAF^39^. Direct AF termination during CFAE ablation was most frequently observed in RA, including the atrial septum^40^. In our study, we demonstrate, using bi-atrial DTs, that the RA is a major arrhythmogenic substrate with considerable fibrotic remodeling, capable of giving rise and sustaining AF drivers (rotors).

Our approach has a strong focus on lesion minimization to preserve atrial function. Substrate ablation outside PVI in an ad hoc fashion has not only shown no improvement in freedom from PsAF^6,7^ but also has led to frequent occurrence of scar-related iATs and, less commonly, stiff LA syndrome due to excessive ablation^25,26^. To avoid unnecessary substrate ablation, an approach to lesion minimization that accounts for the local arrhythmogenic properties of the substrate needs to be developed first. Second, it needs to be established whether a proposed lesion has a low or a high likelihood of developing iAT, so that ablation lines are drawn, in case of high likelihood, connecting those lesions to non-conductive barriers. Minimizing linear lesions for iAT control is an essential step, as these lesions not only decrease atrial function but also often result in the formation of gaps in the lines even with high power ablation, leading to new reentries after ablation^41^. These two strategic steps are achieved here using our DT technology. Note that, although we previously used computational models of patients’ atria to guide ablation in patients with PsAF, the models aimed only to achieve substrate non-inducibility without considering the clinically important issue of lesion minimization^27^. Compared to previous such approaches, our strategy achieves a substantial decrease in the amounts of (1) lesions targeting rotor locations (34%) and (2) iAT-preventing linear lesions (47%) in our patient cohort.

Our approach underscores the importance of precisely identifying LIRs and LCRs, which cannot be done intra-procedurally. Previously, attempts have been made to distinguish active AF rotational activities (drivers) from passive ones so that the former can be targeted^19–22,42^. It is important to note that, in our DTs, there are passive rotational activities that occur continuously as waves emanate from a persistent rotor (both LIR and LCR are persistent rotors). Our study goes mechanistically beyond the current driver classification; it uncovers that even sustained rotors (drivers) can differ in their capability to persist in a substrate that is being modified.

We also explored the features of intra-procedural electrograms recorded at rotor locations to find out whether they reflected the substrate features found essential in lesion-minimizing targeting. In previous studies, endocardial EAM-based substrate characteristics, such as CFAE and LVA, have been used in PsAF ablation^14,31^; however, meta-analyses reported no effectiveness of targeting them^6,7,16^. Recently, FAAM was reported to be an indicator of vulnerable substrates and an effective target for PsAF ablation^43–45^. In our study, the recorded endocardial EAMs could not discern LCRs from LIRs, but the FAAM potential was found useful for predicting iAT locations.

Here we demonstrate that DTs are a valuable tool in exploring the atrial arrhythmogenic substrate, rotor sustainability and the influence of one rotor on another. Our DT sequential simulation of rotor location targeting reveals that LCRs have less power to sustain rotors in a substrate modified by ablation, thereby narrowing down LIRs as ablation targets and establishing an optimal ablation strategy. Our approach is transformational not only because it minimizes lesions but also because it prevents emergent rotors, captured after repeat inducibility tests, thereby minimizing the likelihood of redo procedures. Moreover, we found that the fibrosis distribution visualized by LGE-MRI is helpful in predicting the ablation targets, the LIRs. At least areas with low FD could be excluded from being targeted to avoid unnecessary lesions.

In conclusion, in this combined prospective clinical and computational study, we used DT technology to develop a new lesion-minimizing strategy for ablation in patients with PsAF with fibrotic remodeling that is based on the mechanistic exploration of the atrial substrate. Our results resolve the debate regarding what are the appropriate substrate targets for extra-PVI ablation in these patients. Our approach is designed to preserve atrial function while minimizing the potential for redo procedures. This non-invasive assessment and target prediction could be available before the clinical procedure and used effectively as intraoperative information to guide ablation.

This study has several limitations. First, the personalized DTs are associated with a number of uncertainties stemming from the large number of parameters involved in this type of computational modeling. Although the current study provides a mechanistic assessment of the interaction of the fibrotic substrate with ablation lesions in the DTs and proposes how to exploit these interactions to devise an optimal ablation strategy, it is not a clinical study assessing the utility of the proposed ablation approach in patients with PsAF. For that, prospective studies and, potentially, a clinical trial will need to be conducted.

An additional limitation of this study is the small number of prospective patients. However, many ablation simulations (over 200) were executed in each personalized DT to demonstrate achieving substrate non-inducibility, with over 9,000 simulations of rotor induction tests in total. Another limitation is that all personalized DTs had the same atrial wall thickness. Although this is consistent with previous MRI studies reporting an overall mean wall thickness^46^, we expect that future developments and improvements in digital twinning will advance the technology and potentially improve model construction.

## Methods

### Prospective observational clinical study

This study complies with all relevant ethical regulations and was approved by an institutional review board at Johns Hopkins University (approval number IRB00183327), and informed consent was obtained from each patient. Our imaging-based atrial DT ablation strategy is for patients with AF with fibrotic remodeling, and, thus, as eligibility criteria, we used a cohort of patients with PsAF in whom fibrosis is much more prevalent than in patients with paroxysmal AF. Thirty-five consecutive patients with PsAF who enrolled in this prospective study from 2020 to 2023 underwent cardiac MRI (CMR) examinations before catheter ablation at the Johns Hopkins Hospital. The patient population included seven female patients, and the average age was 64 years. Twenty-six personalized atrial DTs were constructed using LGE-MRI images. Six patients were excluded from the study owing to poor quality of images; three patients with low fibrosis burden (below 5%) were also excluded. As the latter patients had virtually no fibrosis, their AF was likely due to triggered activity from the PVs; they were not appropriate for our research, as it focuses on the arrhythmogenic propensity of the fibrotic substrate. Among 26 DTs, five patients did not have rotors outside of the standard PVI lesion sets. Table 1 summarizes the characteristics of the 26 patients whose personalized atrial DTs were created. Nineteen patients (73%) were male. All patients had enlarged LA and RA. Notably, the mean of body mass index (BMI) was over 30 kg m^−2^ in the cohort, and 15 patients (58%) were obese, with a BMI of over 30 kg m^−2^. Details of previous ablation procedures of patients with repeat ablation are presented in Supplementary Table 3.

### CMR study

CMR examinations were performed 2–3 weeks before the catheter ablation procedure using a 1.5 Tesla MRI scanner (Aera, Siemens Healthineers) (Fig. 1, blue panel, left). To optimize image quality, patients with PsAF were kept on anti-arrhythmic medications and/or referred for cardioversion before CMR. The CMR examination was performed using the same methodology regardless of the presenting rhythm. The CMR protocol included contrast-enhanced magnetic resonance angiography and three-dimensional (3D) LGE-MRI scans. Time-resolved contrast-enhanced magnetic resonanceMR angiography for assessment of LA and PV anatomy was acquired using TWIST (Siemens Healthineers) pulse sequence during intravenous administration of 0.2 mmol kg^−1^ gadolinium-based contrast agent (gadobutrol, Bayer Healthcare Pharmaceuticals). The patients were instructed to breath regularly without deep inspirations/expirations during the atrial 3D LGE-MRI scan. Patient respiration was observed during the respiratory-navigated magnetic resonance angiography scan acquired before 3D LGE-MRI, and the navigator parameters for 3D LGE-MRI scan were adjusted according to the observed patient respiration pattern. The typical scan parameters for the TWIST scan were as follows: coronal imaging volume, repetition time (TR) of 2.23 ms, echo time (TE) of 0.92 ms, flip angle of 25°, in-plane resolution of 0.96 × 1.92 mm, slice thickness of 3.0 mm and reconstructed voxel size of 0.96 × 0.96 × 1.5 mm. The 3D LGE scan for evaluation of LA fibrosis was initiated 20–25 min after contrast agent administration using a fat-saturated 3D inversion recovery-prepared fast spoiled gradient-recalled echo sequence with respiratory navigation and ECG gating. Data acquisition was restricted to 15% of the R-R interval and was performed at end of diastole just before atrial kick. The typical scan parameters for 3D LGE-MRI scan were transverse imaging volume covering LA and RA, TR/TE = 5.2/2.24 ms, flip angle of 20°, in-plane resolution of 1.25 × 1.25 mm, slice thickness of 2.5 mm and reconstructed voxel size of 0.625 × 0.625 × 1.25 mm. Trigger time and data acquisition duration (the number of segments) were optimized to acquire imaging data during the diastole of LA as dictated by inspection of the cine images. The optimal inversion time (TI) for 3D LGE was selected using a TI scout scan of the left ventricle.

### Personalized bi-atrial DTs

**Extended Data Fig. 5 |:**
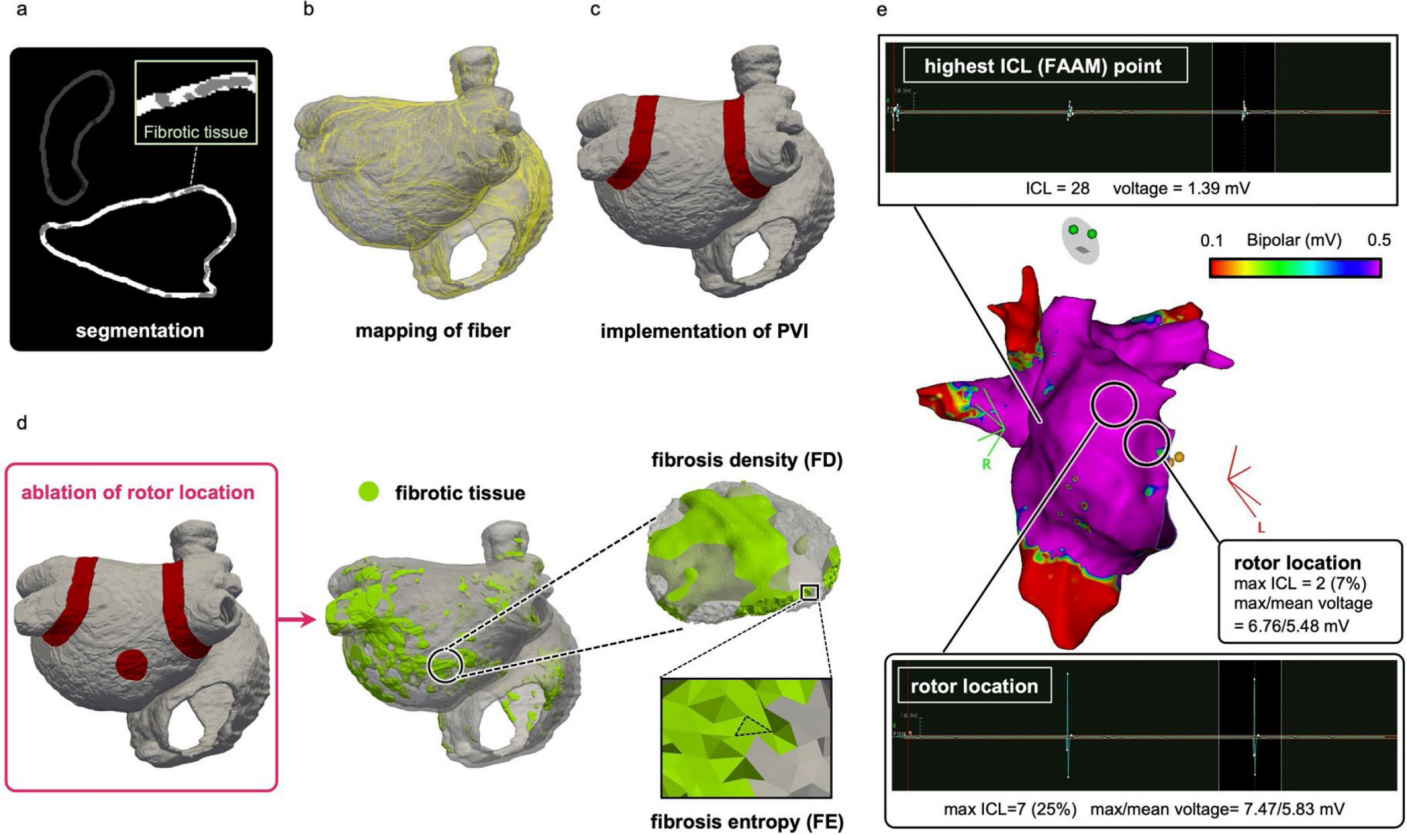
Personalized atrial DTs. **a**: Bi-atrial segmentation of LGE-MRI images with fibrotic tissue in gray. **b**: Fiber mapping (yellow lines) in a DT. **c**: Implementation of PVI ablation lines (red). **d**: Calculation of FD and FE at ablated rotor locations (red circle). **e**: Top image: Voltage amplitude and FAAM potentials of bipolar electrograms with the highest ICL percentage recorded at the mapping point. Middle image: An EAM map created using the CARTO system. Bottom image: Voltage amplitude and FAAM potentials of bipolar electrograms at rotor locations.

In recent years, computational modeling of the patient’s atria has made substantial advances^27,47,48^. The computational methodology for creating patient-specific DTs of the atria in this study was described in detail in our previous publications^27,33,49–52^, with its utility demonstrated in a proof-of-concept prospective study that successfully used DTs to guide ablation^27^. Specifically, 3D fibrosis maps were created (Extended Data Fig. 5a) by semi-automatic segmentation of LGE-MRI scans after identification of the atrial region of interest by CLARAnet, a convolutional deep neural network^53,54^. In addition, using the image-processing software ITK-SNAP (http://www.itksnap.org), quality control was performed manually to standardize the anatomy by detecting potential non-anatomical holes and extra-septal connections.

**Extended Data Fig. 6 |:**
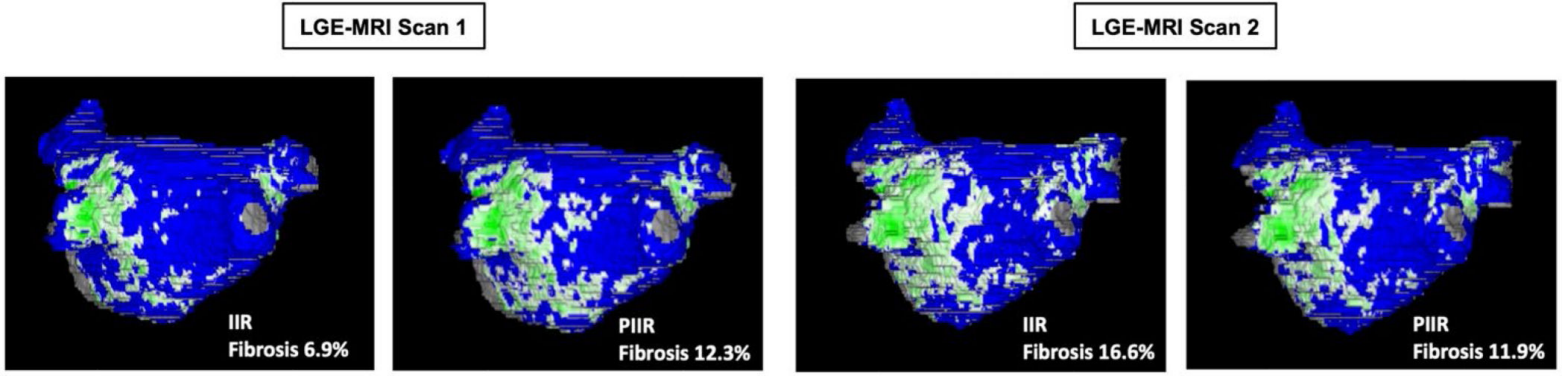
Reproducibility of LA fibrosis assessment using IIR and PIIR methods. The patient had two atrial 3D LGE scans during the same MRI session. Each scan was independently segmented by two experienced operators. IIR method with the widely used IIR threshold of 1.22 gave considerably different fibrosis values (6.9% and 16.6%) for scan 1 and 2. Whereas, the PIIR method gave similar fibrosis values (12.3% and 11.9%) for both scans. The PIIR threshold for scan 1 was 1.164 and the PIIR threshold for scan 2 was 1.263.

Fibrotic and non-fibrotic tissue regions were differentiated using a novel image intensity ratio (IIR)-based approach developed specifically for this study. The conventional IIR approach^55^ based on the intensity of atrial wall and blood pool depends on multiple factors: field strength of scanner, contrast agent type and dose, delay between contrast administration and 3D LGE scan, heart rate, patient-specific contrast clearance rate and hematocrit and blood oxygenation levels. To avoid underestimating or overestimating fibrosis, a personalized thresholding approach (personalized IIR (PIIR)) for atrial fibrosis was applied in this study, using two reference tissues (atrial blood pool and aorta wall, the latter composed entirely of fibrous tissue) instead of one reference (atrial blood pool) used in conventional IIR. The normalization of atrial IIR by mean aorta wall IIR minimizes the effects of the above-mentioned factors on atrial fibrosis threshold. The new PIIR approach has markedly better reproducibility in fibrosis assessment from repeated 3D LGE scans than conventional IIR (Extended Data Fig. 6). In this study, the PIIR threshold of fibrotic tissue provided by a historical cohort was computed for each patient, and voxels with IIR above this value were classified as fibrotic tissue.

Next, after generating the fibrosis map, the segmented and carved LGE-MRI was transformed into a 3D tetrahedral mesh generated by Materialise Mimics (https://www.materialise.com), with a target edge length of 300 μm (Fig. 1, top left image within the light blue box). Fiber directions were computed and mapped from diffusion tensor imaging data^56^ onto patient-specific meshes using a custom 3D mapping software and the Universal Atrial Coordinates approach^57^ (Extended Data Fig. 5b). The methodology for digital twinning the electrophysiology of the atria of patients with PsAF with fibrotic remodeling can also be found in our previous publications^27,49,50,58^. In brief, we used a human chronic AF atrial action potential model modified to fit clinical monophasic action potential recordings from patients with AF to represent membrane kinetics in non-fibrotic regions. In fibrotic regions, additional modification to the action potential model were implemented to represent the effect of elevated transforming growth factor β1 associated with the activated pro-fibrotic signaling pathway, by reducing maximal *I*_K1_, *I*_CaL_ and *I*_Na_ channel conductances by 50%, 50% and 40%, respectively. The single-fiber non-fibrotic longitudinal-to-transverse anisotropy ratio was 5:1, whereas the fibrotic was 8:1. The non-fibrotic longitudinal conduction velocity, when tested on planar propagation in a tissue slab, was 43.39 cm s^−1^, whereas the fibrotic was 20 cm s^−1^. Typical reentrant frequency of our model was 3.6 Hz. Finally, all simulations were executed on a parallel pipeline computing system^59^ using the freely available (for academic purposes) openCARP software (https://opencarp.org)^60^.

### Simulation of ablation in the atrial DTs

As described in our previous studies, ablation lesions in the personalized DTs were represented by replacing myocardial tissues with scar (non-conductive) tissue^27^. Linear lesions representing PVI, executed first after model creation, were of width 7.5 mm. Ablation lesions at LIRs or LCRs were of diameter 12 mm on the surface (Extended Data Fig. 5c,d), extending transmurally in a hemispherical fashion. These lesions are larger than single-point lesions, as they need to encompass any meander of the induced rotor; in a clinical procedure, they would entail executing several single-point ablations. When necessary, ablation lines were extended from the LIR/LCR lesion to a non-conductive barrier, such as mitral valve or PVI line, to prevent iAT occurrence.

### Assessment of arrhythmogenicity in the DT atrial substrate: rotor induction, detection and classification

**Extended Data Fig. 7 |:**
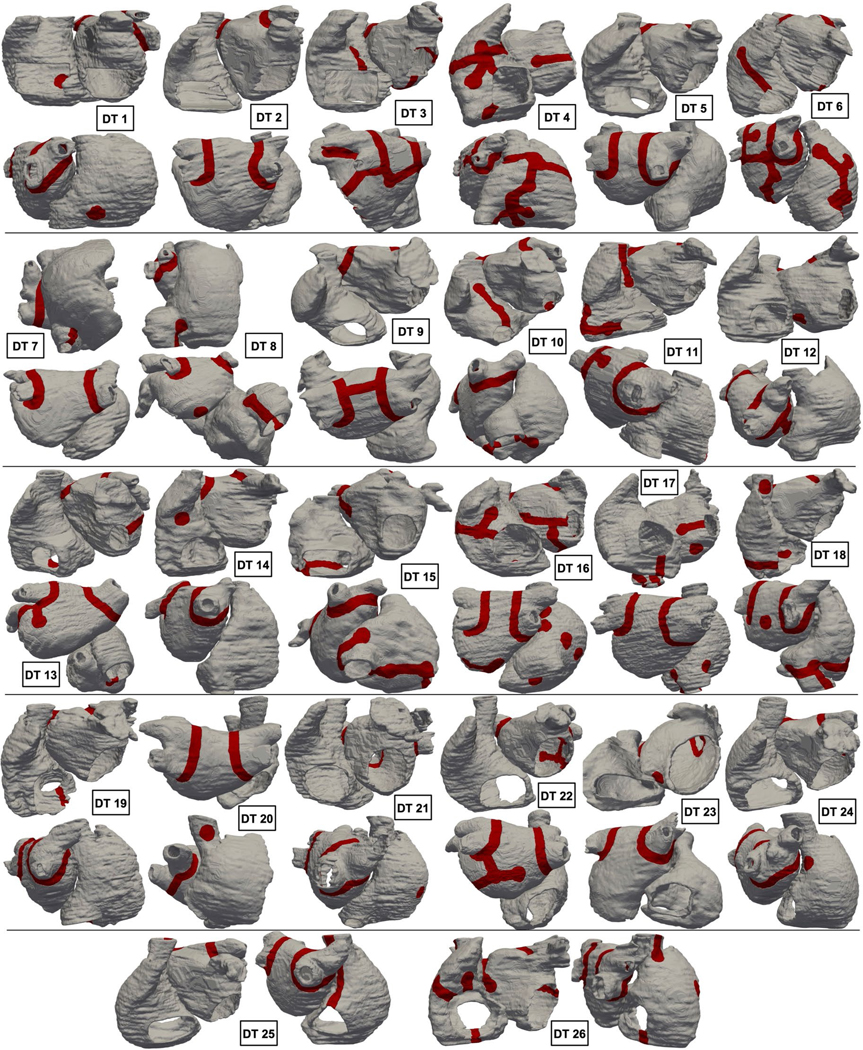
Final lesion-minimizing ablation sets for the patients in the cohort. Lesions are shown in red.

Extra-PVI substrate inducibility tests were performed in the personalized bi-atrial DTs by incremental burst pacing from a distributed set of 40 sites, validated in our previous studies^27,61^; these represented the potential triggers in the fibrotic substrate outside of the PVI lines. Rotational activities (rotors) persisting for over 5 s were detected by examining phase singularity trajectories using freely available Meshalyzer software (https://github.com/cardiosolv/meshalyzer) (Fig. 1, top right image within the light blue box). A rotor-attracting location was defined as the center of the phase singularity trajectory area, and ablation centered at it. Most rotors were stationary or meandering in a small area, but 10 rotors widely meandered. In these cases, the lesions were extended, covering ellipses with a minor axis diameter of 12 mm and a major axis in the direction of meandering (21 ± 4 mm). Rotor-attracting locations were classified as belonging to 12 regions: anterior wall of the LA, posterior wall of the LA, LA roof, inferior LA, posterolateral LA, atrial septum, anterior RA wall, posterior RA wall, inferior RA wall, lateral RA wall and areas near the superior and inferior vena cavas. All extra-PVI rotor-attracting locations induced in the substrate were investigated one by one to determine whether ablating them would result in iAT and would require a linear ablation connecting the lesion to a non-conductive barrier (Fig. 1, orange panel within the light blue box). Next, to achieve rotor non-inducibility in each atrial DT, a large number of ablation strategies to eliminate locations in the substrate capable of sustaining rotors were simulated and examined sequentially as guided search. In this guided search, the effect of ablation of the location of each individual rotor on the occurrence of other rotor-attracting locations was investigated. Substrate locations attracting rotors were classified into two types: a location where the rotor became non-inducible in the substrate after ablation of another rotor location was defined as an LCR, whereas a location where the rotor persisted unaffected by the ablation of another rotor-attracting location and needed to be ablated to achieve non-inducibility of the fibrotic substrate was defined as an LIR. The LIRs were prioritized for ablation in the DTs, and, after ablation, the DT inducibility test was performed again. This process was repeated until the substrate was no longer capable of giving rise to rotors after rapid pacing from any bi-atrial site (Extended Data Fig. 7).

Simulations were performed blinded to the EAM data. The investigation of the EAM data and the analysis of the simulation data were performed only after the simulations were completed.

### Analysis of LGE-MRI fibrosis distribution

Fibrosis distribution was evaluated using the FD and FE measures of fibrosis distribution (Extended Data Fig. 5d), which were used to assess arrhythmogenic propensity in our previous study^33^. A 3D map of FE distribution, indicating the level of fibrosis structural disorganization, was constructed in each bi-atrial DT based on the FE value at a given location as well as on the FE values at surrounding locations within a 2.5-mm radius, a kernel size equal to the resolution of LGE-MRI, as done in our previous publication^33^. Then, the regional FE value at each rotor location (ablated or not) was calculated by averaging the 3D FE values within the area where the rotor persisted. Next, the FD value at each rotor location was calculated as the ratio of the total volume of mesh elements with enhancement (fibrosis) to that of all mesh elements within that rotor location.

### Analysis of intra-atrial electrograms

One patient did not undergo catheter ablation; in the simulations for this patient, there were no rotors induced outside of PVI. Only one patient underwent electroanatomical mapping during AF due to cardioversion-refractory AF, and, therefore, three rotor locations mapped in this patient were excluded from EAM analysis. Some patients underwent no mapping of RA because of procedure time limitations or clinician preference.

After placing transseptal sheaths in the LA via the femoral vein, mapping of intra-atrial electrograms was done using a multi-electrode mapping catheter (PentaRay, Biosense Webster) and an EAM system (CARTO-3, Biosense Webster). When the rhythm was SR, the bipolar voltage map and the FAAM map were analyzed, and the difference between LIRs and LCRs or between iAT-inducing locations and iAT-free locations was investigated^43^. The mean of the bipolar voltage at a rotor location was calculated by averaging the bipolar voltage at each mapping point at that location. The highest bipolar voltage among mapping points within the rotor location was defined as the maximum of bipolar voltage at the rotor location. Intra-atrial bipolar electrograms with intervals from 0 ms to 10 ms and amplitudes of 0.1 mV to the highest voltage amplitude within the whole atrium were defined as FAAM segments in SR. Then, the ratio of the number representing the ICL in each rotor location to the highest number of ICLs within the whole atrium was determined as the ICL percentage (Extended Data Fig. 5e). Noisy potentials due to poor contact to the atrial wall or movement of the catheter, interruption by premature atrial contractions or oversensing of ventricular potential near the valve were excluded from ICL analysis.

### Statistical analysis

Numerical variables are represented as the mean ± s.d. and the median (Q1–Q3) for normally and non-normally distributed data, respectively. Normal distribution was confirmed using a histogram and the Shapiro–Wilk normality test. Tests for statistical significance were performed using *t*-tests or Mann–Whitney *U*-tests depending on distribution. Categorical variables are represented as the *n* (%) and were compared using chi-square tests or Fisher’s exact tests. Sensitivity, specificity and accuracy for LIRs and locations of iAT occurrence were calculated using receiver operating characteristic (ROC) curves and AUCs to determine the cutoff for FD, FE and ICL percentage. The cutoff value was determined so that the sum of sensitivity and specificity was maximized. Linear regression analysis was performed to assess independent association between fibrosis burden and clinical parameters and that between the ICL percentage and fibrosis distribution or EAM bipolar voltage. Any variable having *P* < 0.1 in the univariate analysis was included in the multivariate models. Correlation analysis between the number of rotor locations or LIRs and fibrosis burden or atrial volume was performed using Spearman’s rank test. A response variable was natural log transformed in case of no normality in the regression residuals. *P* < 0.05 was considered statistically significant for all tests.

All statistical analyses were conducted with EZR software, which is a graphical user interface for R (R Foundation for Statistical Computing). We used a modified version (1.55) of R Commander designed to add statistical functions frequently used in biostatistics.

### Reporting summary

Further information on research design is available in the Nature Portfolio Reporting Summary linked to this article.

### Data availability

Data supporting the findings of this study are available within the paper and its Supplementary Information. Patient MRI data used to construct the personalized digital twins are available upon reasonable request and upon approval from the Johns Hopkins institutional review board.

### Code availability

The image-processing software ITK-SNAP is freely available from http://www.itksnap.org. Computational meshes were generated using the commercial 3-matic analysis software (Materialise Mimics). All simulations were performed using the freely available openCARP software for academic purposes from https://opencarp.org. Simulation results were visualized using the freely available software Meshalyzer from https://github.com/cardiosolv/meshalyzer or the open-source Visualization Toolkit–based software ParaView from https://www.paraview.org. Ablation data were analyzed using the commercial EAM system (CARTO-3, Biosense Webster). Computational meshes and the parameter files required for simulations with openCARP, the source data used to produce analysis in Fig. 3 based on the simulation results, and the data and codes for the analysis shown in Figs. 2, 5 and 6 are available at https://gitlab.com/natalia-trayanova/assessing-the-arrhythmogenic-propensity-of-fibrotic-substrate-using-digital-twins.

## Extended Data

## Supplementary Material

Supplementary Table 2

Supplementary Table 1

Supplementary Table 3

STROBE-checklist

## Figures and Tables

**Table 1 |:** Baseline patient characteristics

	*n* = 26
Male	19 (73)
Age, years	65 ± 12
BMI, kg m^−2^	31 ± 5
CHA_2_DS_2_-VAS_c_ score	2.2 ± 1.2
LVEF, %	54 ± 9
LA volume, ml	145 ± 43
RA volume, ml	144 ± 38
LA and RA volume, ml	289 ± 69
LA fibrosis burden, %	16 ± 6
RA fibrosis burden, %	14 ± 8
Total atrial fibrosis burden, %	15 ± 6
Days from MRI study to ablation, days	20 ± 8
No effective cardioversion and/or anti-arrhythmic drugs	14 (54)
Previous atrial ablation	13 (50)

Values are mean ± s.d. or *n* (%). LVEF, left ventricular ejection fraction.
